# Bilateral Lacrimal Gland Lymphoma in Sjögren Syndrome

**DOI:** 10.1155/2016/2798304

**Published:** 2016-09-21

**Authors:** Melis Palamar, Nazan Ozsan, Fahri Sahin

**Affiliations:** ^1^Department of Ophthalmology, Faculty of Medicine, Ege University, Izmir, Turkey; ^2^Department of Pathology, Faculty of Medicine, Ege University, Izmir, Turkey; ^3^Department of Hematology, Faculty of Medicine, Ege University, Izmir, Turkey

## Abstract

A 31-year-old female with Primary Sjögren Syndrome (pSS) presented with bilateral puffiness around the eye for 3 years. The lacrimal glands were hypertrophic and edematous bilaterally. Schirmer 1 score was 2 and 1 mm and tear-film break-up time was 3 and 4 seconds, in the right and the left eyes, respectively. An incisional biopsy from the left lacrimal gland revealed diffuse and intense CD20, CD5, and bcl-2 positivity with negative cyclin D1 and CD23 which supported lymphoma. Upon haematology consultation extranodal marginal zone lymphoma diagnosis was made. CHOP (cyclophosphamide, doxorubicin, vincristine, and methyl prednisolone) treatment was initiated. In conclusion, pSS is a well known autoimmune disease in which increased rate of lymphoma is present. Early detection with histopathologic confirmation and multidisciplinary approach with ophthalmology, rheumatology, and haematology are mandatory in these patients.

## 1. Introduction

Sjögren syndrome is a chronic autoimmune condition which goes along with lymphocytic and plasma cell infiltration of the salivary and lacrimal glands progressively. This gland infiltration finally leads to xerostomia and keratoconjunctivitis sicca [1]. Sjögren syndrome might be either primary or accompanying other autoimmune diseases [1]. It is well known that, in patients with Sjögren syndrome, lymphoproliferative activity, benign to malign, is significantly increased [2, 3]. In Sjögren syndrome the relative risk of lymphoma is estimated to be 16–18 times higher than the normal population [4, 5]. Herein we report bilateral lacrimal gland lymphoma in a female with Primary Sjögren Syndrome (pSS).

## 2. Case Presentation

**Figure 1:**
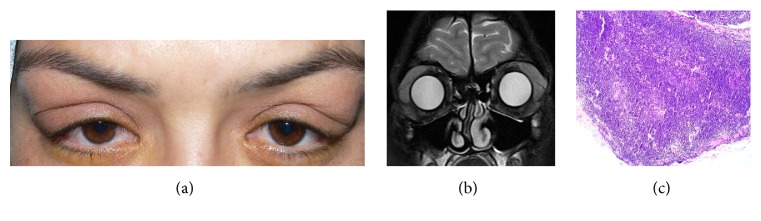
(a) Bilateral puffiness in lacrimal gland region is evident. (b) On MRI bilateral hypertrophic lacrimal glands are seen. (c) Histopathologic examination revealed neoplastic infiltration of small to medium sized lymphoid cells forming a vaguely nodular pattern (H&E, ×10).

A 31-year-old female with pSS for 3.5 years experiencing bilateral periorbital puffiness for 3 years was evaluated. Upon inspection lacrimal gland regions were bilateral puffy (Figure 1(a)). Eversion of the eyelids led the view of hypertrophic and edematous lacrimal glands. Best corrected visual acuity was 20/20 in both eyes with normal anterior and posterior segment evaluation. Schirmer 1 score was 2 and 1 mm and tear-film break-up time was 3 and 4 seconds, in the right and the left eyes, respectively. On magnetic resonance imaging both of the lacrimal glands were hypertrophic (Figure 1(b)).

**Figure 2:**
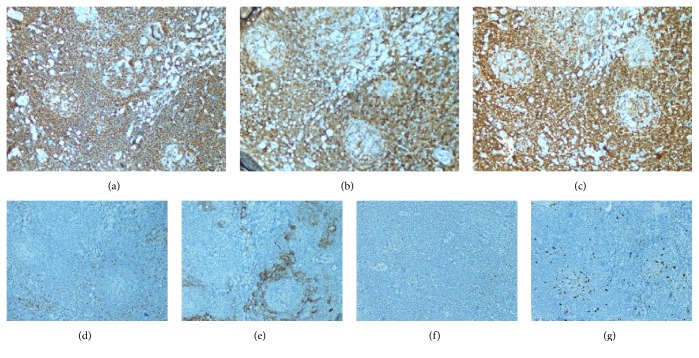
Immunohistochemically (×20), neoplastic infiltration was positive with CD20 (a), CD5 (b), and Bcl-2 (c) but negative with CD3 (d), CD23 (e), and cyclin D1 (f) and Ki67 revealed low proliferation index (g).

An incisional biopsy from the left lacrimal gland, which was more hypertrophic, was performed. Haematoxylin-eosin staining revealed neoplastic infiltration of small to medium sized lymphoid cells forming a vaguely nodular pattern (Figure 1(c)). Immunohistopathologic evaluation revealed diffuse and intense CD20, CD5, and bcl-2 positivity with negative cyclin D1 and CD23 (Figures 2(a)–2(f)). The results supported lymphoma; however, the exact histological discrimination could not be made. The patient was consulted to haematology department and as lacrimal gland was affected extranodal marginal zone lymphoma was diagnosed. PET/CT scan was performed for staging. PET/CT scan showed pathological uptake FDG-18 in bilateral cervical lymph nodes and clinical stage (Ann Arbor) was IIE. CHOP (cyclophosphamide 750 mg/m^2^ D1, doxorubicin 50 mg/m^2^ D1, vincristine 2 mg D1, and methyl prednisolone 100 mg/d D1-5) treatment was initiated. The patient received 6 cycles of CHOP. Complete remission (CR) was achieved after 4 cycles of the treatment. The patient maintained a CR for 12 months.

## 3. Discussion

The diagnosis of lymphoma in Sjögren syndrome is well documented and lymphoma is recognized as the most important complication in the disease progress [4]. The evolution from the lymphoepithelial disease to lymphoma is likely to be a multiphase activity [6]. Lymphoepithelial disease is mostly seen as a compound of polyclonal B and T cells, whereas lymphoma shows neoplastic monoclonal B-cell proliferation [3]. An intermediate stage lymphoproliferative activity in the spectrum of benign to malignant lymphocytic proliferation is accepted as pseudolymphoma [5].

In patients with Sjögren syndrome various histologic subtypes of lymphoma, most of which are low-grade B-cell malignancies, have been described [7]. It is reported that mucosa associated lymphoid tissue lymphoma constitutes 46 to 56% of the lymphomas, developing in Sjögren syndrome patients [7].

The higher risk of developing lymphoma in Sjögren's syndrome is predicted to increase with time of the diagnosis. However, the risk is reported not to be related to the patient age at pSS diagnosis and is reported to be less among patients without any bad predictive factors [5]. It is advised that whenever asymmetrical enlargement of parotid or lacrimal glands, palpable masses in the glands, refractory lymphadenopathy, and splenomegaly are detected, lymphoma should be suspected and investigated. Herein, the diagnosis time of pSS was 3.5 years earlier and bilateral puffiness around lacrimal gland region was experienced for 3 years. What makes this case unique is the bilateral lymphoma involvement of the lacrimal glands in pSS, to our knowledge the first one in the literature.

Treatment options for marginal zone lymphoma (MZL) are considered when patients have limited stage, advanced stage, and location of disease. Although patients with limited stage MZL are managed with radiotherapy (24–36 Gy), rituximab monotherapy and rituximab combined with chemotherapy need to be evaluated. Rituximab-CHOP combination therapy is the standard treatment option in advanced stages [8, 9]. As the reimbursement agency does not pay for rituximab in this clinical state, we treated the patient with only CHOP regimen.

In conclusion, rate of lymphoma in patients with pSS is markedly increased. For this reason, imaging techniques especially focusing on the head and neck regions including the lacrimal glands are sine qua non in the follow-up of pSS patients. Any enlargement of the lacrimal glands even if bilateral should be investigated for possible lymphoproliferative disease.
